# Mechanism-Based Sonodynamic–Chemo Combinations against Triple-Negative Breast Cancer

**DOI:** 10.3390/ijms23147981

**Published:** 2022-07-20

**Authors:** Xiaolan Feng, Chen Wu, Wenhao Yang, Jiayi Wu, Pan Wang

**Affiliations:** 1 Key Laboratory of Medicinal Resources and Natural Pharmaceutical Chemistry, Ministry of Education, Xi’an 710119, China; fengxlsd@snnu.edu.cn (X.F.); wuchen029@163.com (C.W.); ywhzxc@snnu.edu.cn (W.Y.); jiayiwu33@163.com (J.W.); 2 National Engineering Laboratory for Resource Developing of Endangered Chinese Crude Drugs in Northwest of China, College of Life Sciences, Shaanxi Normal University, Xi’an 710119, China

**Keywords:** sonodynamic therapy, porphyrin liposomes, DOX, SRA737, DNA damage response, breast cancer

## Abstract

Due to its noninvasive nature, site-confined irradiation, and high tissue penetrating capabilities, ultrasound (US)-driven sonodynamic treatment (SDT) has been proven to have broad application possibilities in neoplastic and non-neoplastic diseases. However, the inefficient buildup of sonosensitizers in the tumor site remarkably impairs SDT efficiency. The present work proposes a deep-penetrating sonochemistry nanoplatform (Pp18-lipos@SRA737&DOX, PSDL) comprising Pp18 liposomes (Pp18-lipos, Plipo), SRA737 (a CHK1 inhibitor), and doxorubicin (DOX) for the controlled formation of reactive oxygen species (ROS) and release of DOX and SRA737 upon US activation, therefore increasing chemotherapeutic effectiveness and boosting SDT efficacy. Therein, the antitumor activities of DOX have been attributed to its intercalation into the nucleus DNA and induction of cell apoptosis. CHK1 evolved to respond to DNA damage and repair the damage via cell cycle progression. SRA737 is a potent and orally bioavailable clinical drug candidate for inhibiting CHK1, demonstrating adjuvant anticancer effect in vitro and in vivo. It was interesting to find that SRA737 carried into Plipo@DOX could significantly alleviate G2/M cell cycle arrest and aggravate DNA double-strand injuries, resulting in significant cell death. The developed US-switchable nanosystem provides a promising strategy for augmenting sono-chemotherapy against breast cancer controllably and precisely.

## 1. Introduction

Triple-negative breast cancer (TNBC) is the most challenging subtype that frequently develops resistance or non-response to the backbone of chemotherapy [1]. During the past decade, great effort has been devoted to classifying TNBC into distinct subtypes which can guide treatment decisions [2]. Meanwhile, the new appreciation of the biology of TNBC is accelerating the development of novel drugs, including antibody–drug conjugates, PARP inhibitors, and most recently immune-checkpoint inhibitors for patients with early-stage TNBC and for those with advanced-stage disease [3]. However, in many cases, unwanted side effects lead to a high failure rate of therapy. A reduced treatment dose is often necessary to limit side effects caused by untargeted agents. Purushottamachar et al. recently synthesized three novel deuterated analogs that can be efficacious against TNBC cell proliferation using a dramatically lower dose than the nondeuterated compound. However, whether these chemotherapeutic drugs may finally lead to cancer recurrence remain unknown [4]. In this case, some external stimulus means such as heat [5], light [6], magnetism [7,8], and ultrasound (US) [9] have been investigated to aid topical drug administration, and they are all practicable for clinical and research applications. US is typically used to stimulate sound-sensitive agents (sonosensitizers) to exert cytotoxic effects, a process referred to as sonodynamic therapy (SDT). SDT is a promising alternative to conventional cancer therapy due to its noninvasiveness, superior tissue penetration capability, and site-confined irradiation provided by US [10].

In the SDT process, sonosensitizers can remarkably influence the effects of US by inducing ROS-dependent cytotoxicity during US stimulation. Generally, the hydrophobic nature and self-aggregation of sensitizers in the physiological environment affect their pharmacokinetic properties and attenuate the ultrasonic response. To circumvent these constraints, nanotechnology in conjunction with sensitizers has shown tremendous promise in the US-mediated diagnosis and therapy of cancer [10,11,12,13]. By dropping the cavitation threshold, the coated nanoparticles can offer more nucleation sites for cavitation bubble formation, hence increasing SDT efficiency [14]. In our previous works, a liposome-like nanoporphyrin (Pp18-lipos) self-assembled from lipid–purpurin 18 conjugates (Pp18-lipids) and pure lipids has been designed. In detail, nanoporphyrin with a low density of Pp18 not only can generate ROS upon exposure to low-intensity ultrasound irradiation for SDT to destroy cells directly [15,16], but also can be used as a carrier loaded with other therapeutic drugs to achieve combination therapy.

Doxorubicin (DOX) is an anticancer medication with a broad range of applications in anticancer treatment. The antitumor effects of DOX are thought to be due to its ability to intercalate into DNA and cause DNA damage [17]. DNA damage elicits the activation of multiple signal pathways and launches growth arrest as well as DNA damage-repair machinery [18,19]. Checkpoint kinase 1 (CHK1) is a master modulator of the DNA damage response that controls cell cycle progression, DNA damage repair, and DNA replication, dampening excessive DNA damage and boosting the tumor cells’ overall survival fitness [20]. Furthermore, according to published data from The Cancer Genome Atlas (TCGA) as well as the Genotype-Tissue Expression Program (GTEx) data resources, CHK1 expression in breast cancer is remarkably higher relative to that in vicinal peritumoral tissues [21]. In addition, the TCGA data have also shown a strong link between CHK1 and the ER/PR status. Meanwhile, inhibiting CHK1 causes TNBC to behave differently from ER^+^/PR^+^/HER^2-^ breast cancer in terms of proliferation, apoptosis, and chemoresistance [21]. Such cancer-specific dependency could be exploited for therapeutic gain, making CHK1 an attractive anticancer target.

Considering the prospect of CHK1 inhibition in cancer treatment, multiple inhibitors of CHK1 have been developed to promote DNA-damaging therapies, but they have failed in clinical trials due to various reasons. SRA737 (formerly known as CCT245737), an orally novel ATP docking site CHK1 inhibitor, is currently under clinical trials as a monotherapy or in combination with chemotherapeutic agents, in particular low-dose gemcitabine, in different cancers [22,23], which can result in remarkable increases in the sensitivity of cancer cells to diverse anticancer drugs. In addition, in mouse models of E-MYC-driven B-cell lymphoma and juvenile MYCN-driven neuroblastoma, SRA737 has been documented to be effective as a single agent in vivo [24]. SRA737 has also been illustrated to enhance gemcitabine and SN38 anticancer efficacy in a human tumor xenograft model while lowering toxicity [25]. Following the generation of promising preclinical data, SRA737 deserves further exploration as an adjunctive drug for TNBC cancer therapy.

Therefore, in order to explore the mechanism-based mutually reinforcing sono-chemotherapy for TNBC, we prepared and characterized Pp18-liposomes loaded with SRA737 and DOX (PSDL) and tested the in vitro along with in vivo antitumor efficiency, in which SRA737 would amplify the DOX-induced DNA damage and augment sonochemical effects. The inherent fluorescence of Pp18 could guide the appropriate US exposure window when the nanocomposites accumulated to a higher level in tumors, and the Pp18-US stimulation further released the cargos in situ and rapidly achieved sufficient therapeutic drug concentration in the tumor area, including the deep interior of tumors. Such mechanistically determined combinations are expected to achieve an ideal therapeutic outcome and can solve the problems of high dose and low biosafety that exist in single therapy.

## 2. Results and Discussion

### 2.1. Preparation and Characteristics of PSDL Nanoparticles

**Figure 1:**
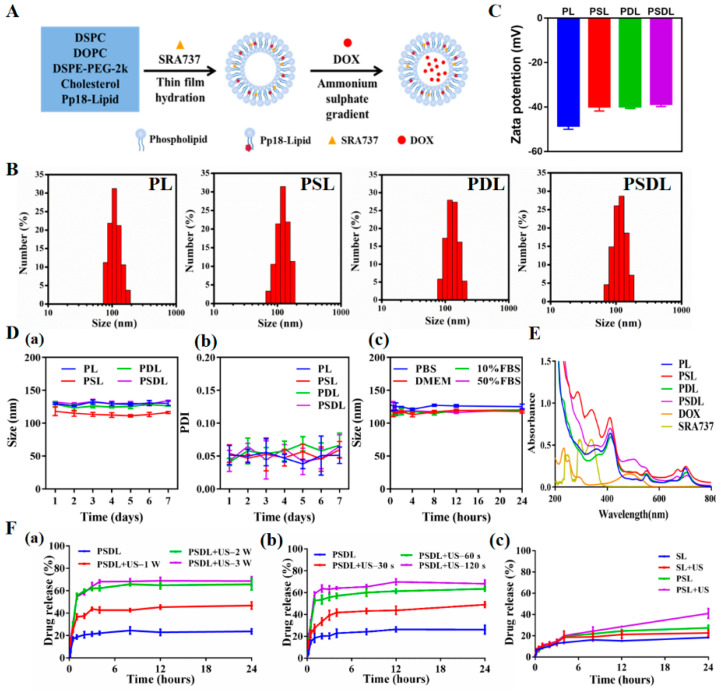
Synthesis and characterization of PSDL. (**A**) Schematic illustration diagram of the PSDL; (**B**) the particle size distribution of different liposomes; (**C**) zeta potentials of each group; (**D**) particle size stability of PL, PSL, PDL, and PSDL at 4 °C (**a**); PDI of PL, PSL, PDL, and PSDL at 4 °C (**b**); the particle size stability of PSDL in PBS, DMEM, 10% FBS, and 50% FBS at 37 °C (**c**); (**E**) UV-Vis absorption spectrum of each group; (**F**) release profiles of DOX from PSDL with/without different ultrasonic intensity (**a**), ultrasonic time (**b**), and the release profiles of SRA737 (**c**) from PSL in the absence and presence of ultrasonic stimulation.

The PSDL synthetic process is schematized in Figure 1A. In the presence of an aqueous environment, amphiphilic phospholipids tend to form liposomes with bilayer structures spontaneously. Unlike polymeric particles, these structures are stable physically and are not covalently bonded. Drugs can be encapsulated in the aqueous core or in the liposome’s surrounding bilayer, depending on the drug’s water solubility [26,27]. Here, the hydrophobic SRA737 was encapsulated inside the lipid membrane while the hydrophilic DOX was integrated into the central aqueous core. Next, the particle size distribution was detected. As shown in Figure 1B, the mean diameters of PL (Pp18-liposomes), PSL (Pp18-liposomes carrying SRA737), PDL (DOX-loaded Pp18-liposomes), and PSDL were ~114 nm, 125 nm, 126 nm, and 132 nm, respectively. Meanwhile, the zeta potential of different liposomes was shown in Figure 1C, which slightly increased from −48.43 to −38.67 mV. The liposomes’ negative charge could help them avoid cross-talking with negative plasma components, improving circulation stability in vivo [28,29]. The size, the polydispersity index (PDI) of these liposomes remained almost the same for seven days, exhibiting good stability (Figure 1D). Next, the absorption and fluorescence spectra of different liposomes were analyzed. As illustrated in parts of Figure 1E, the UV-Vis absorption spectrum of PL revealed three characteristic peaks of Pp18 at ~410 nm, ~550 nm, and ~705 nm, illustrating the insertion of Pp18 in the liposomes. PSL showed two extra absorption peaks: one at 280 nm and the other at 340 nm, which was consistent with the absorption peaks of free SRA737, suggesting the successful load of SRA737 in the liposomes. Similarly, compared to the spectral properties of PL, free DOX and SRS737, the PDL and PSDL were also successfully prepared. Moreover, the spectral peaks with maxima at ~590 and ~705 nm were observed for DOX (Supplementary Figure S1A) and Pp18 (Supplementary Figure S1B) fluorescence of porphyrin liposomes. Notably, the fluorescence values of PSL, PDL, and PSDL exhibited a slight blueshift and change in the height when compared with PL, which reflected that the carried DOX and SRA737 might influence the Pp18 spectral pattern.

### 2.2. Drug Loading and Releasing Properties of PSDL

Improved bioavailability; reduced toxicities; enhanced drug delivery; and the ability to incorporate other functional materials for imaging, sustained release, targeting delivery, and loading more than one drug for combination therapies are all advantages of nanoparticles over free drugs [30]. The drug loading efficiency, which is defined as the mass ratio of drug to drug-loaded nanoparticles, is one of the most important characteristics. Here, the concentration–absorption intensity standard curve of SRA737 and the concentration–fluorescence intensity standard curve of DOX were firstly derived (Supplementary Figure S2A,B). This proportion of SRA737 and DOX in the synthesis process had been optimized to achieve a relatively good loading effect (Supplementary Tables S1 and S2). In addition, the encapsulation efficiencies of the two drugs did not affect each other (Supplementary Table S3). Compared to the application dosage of SRA737 and loading rate of DOX into liposomes by other studies, the Pp18 liposomes prepared herein were co-loaded with SRA737 and DOX, with a relatively satisfactory total drug-loading rate, as well as drug-loading performance.

The cargo contained in the nanoparticles may be released into the solvent as the medium conditions change. The repulsive force between the drugs and the solvent is critical to the release content. Porphyrin liposomes showed US-triggered disruption upon US irradiation. With increasing intensity (Figure 1F(a)) and exposure time (Figure 1F(b)), the sonication process promoted progressive PSDL breakdown, as shown by increased DOX release of roughly 40% to 70%. Of course, the SRA737 had a relatively low drug release because of its limited drug loading (Figure 1F(c)). These results demonstrate that US can achieve efficient drug delivery by conquering physiological barriers and controlling drug release at targeted sites to improve drug bioavailability. In addition, the sono-activatable liposomes combined with chemotherapeutic drugs such as DOX are even more effective against tumor cells. Loading drugs within the capsule, and simultaneous modulation of cargos release are promising in clinical medicine, particularly in medical oncology, and adding functional nanoparticles can further give aid in drug delivery and medical diagnostics [31,32].

### 2.3. In Vitro Antitumor Effect

#### 2.3.1. Ultrasound Enhanced Drug Uptake

**Figure 2:**
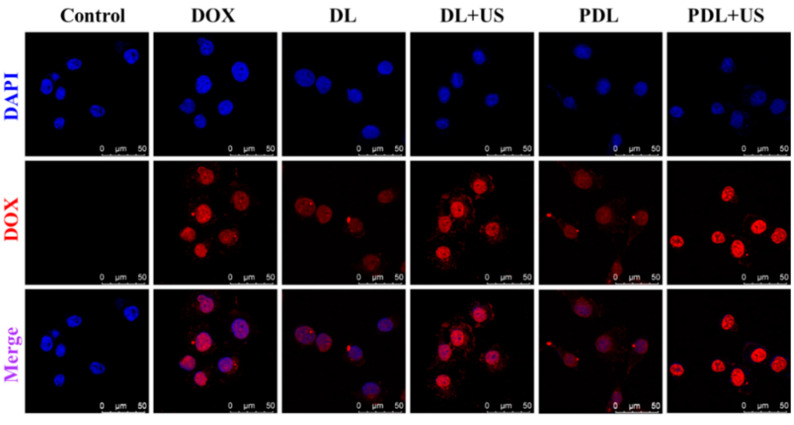
Laser confocal microscope observation of cell uptake of nanoparticles using the red fluorescence emission of DOX (Control: DMEM medium; DOX: free DOX; DL: DOX-liposomes; DL+US: DOX-liposomes + US irradiation; PDL: DOX-loaded Pp18-liposomes; PDL+US: DOX-loaded Pp18-liposomes + US irradiation; scale bar = 50 μm).

As illustrated in Figure 2, after 12 h of inoculation with free DOX, DOX-liposomes (DL) or PDL, free DOX emitted a brilliant red fluorescence and aggregated mostly in the nucleus compartment of the cell. The DL and PDL groups harbored weak fluorescence signals. When used in combination with US, the DL group was more effectively taken up by MDA-MB-231 cells than those used alone. The cells in the PDL group produced a very high fluorescence signal upon US exposure. In this connection, this sonosensitive porphyrin in the liposomes can be able to respond to US to collapse the liposomes, thereby facilitating DOX diffusion and internalization, enhancing DNA damage.

#### 2.3.2. Cell Viability Test

**Figure 3:**
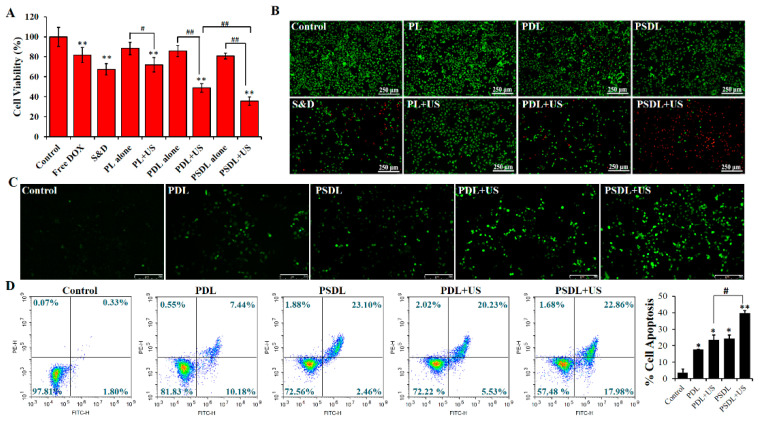
Cytotoxicity evaluation in vitro. (**A**) MDA-MB-231 cell viability after different treatments. (DOX = 1 μg/mL, SRA737 = 0.5 μg/mL; ultrasound: load power 2 W, duration 30 s; S&D: DOX+SRA737); (**B**) detection of MDA-MB-231 cell survival by calcein-AM/PI double staining; (**C**) intracellular ROS generation after different treatments using a fluorescence microscope (scale bar = 250 μm); (**D**) cell apoptosis analysis via annexin V-PI combined with flow cytometry. * *p* < 0.05, ** *p* < 0.01, compared with control; ^#^ *p* < 0.05, ^##^ *p* < 0.01 compared among groups.

To explore the biological effect of US-responsive porphyrin liposomes, MDA-MB-231 cells were exposed to different treatments. Supplementary Figure S3A illustrated that the viability of cells treated with SRA737 or/and DOX was gradually reduced with the increase in drug concentration. As compared with free DOX (0.1, 0.5, 1 μg/mL), there was obvious cell cytotoxicity when the SRA737 level ranged from 0.1 μg/mL to 1 μg/mL. When loaded into PL, the cell cytotoxicity of SRA737 plus DOX was reduced markedly (Supplementary Figure S3B), indicating that liposomes could effectively diminish the toxic and adverse effects of chemotherapy drugs. Then, MDA-MB-231 cells were exposed to different US intensities to investigate an appropriate dose for further evaluation of the biological effects of US-responsive PSDL. Supplementary Figure S3C showed that the killing effect of US on MDA-MB-231 cells gradually decreased with increasing US intensity and time. When US (load power 2 W; duration 30 s) was introduced into porphyrin liposomes, PL, PDL, and PSDL all showed the US-responsive characteristic (Figure 3A). MDA-MB-231 cells treated with PSDL plus US were more sensitized than those treated with PDL combined with US. These findings indicate that the biological effects of synthetic porphyrin liposomes can be regulated through US exposure.

Furthermore, the toxicity of different treatments was further demonstrated via calcein AM/PI staining (Figure 3B). Cells treated with PL or PDL or PSDL alone showed a large portion of green fluorescence with little red fluorescence. However, the DOX plus SRA737 group harbored evident red fluorescence in partial cells, explaining that porphyrin liposomes provided good security by avoiding direct contact and damage to cells caused by chemotherapy drugs. In addition, upon exposure to US, the red fluorescence was gradually enhanced from partial cells to almost all cells after treatment with PL/PDL/PSDL. After that, the increased intracellular ROS level was directly observed using a fluorescence microscope combined with a DCFH-DA probe (Figure 3C). The green fluorescence can be enhanced by increasing intracellular ROS levels. The findings illustrated that cells inoculated with PDL or PSDL alone exhibited only intermittent green fluorescence, with no remarkable difference between them. As expected, the cells harbored bright green fluorescence after the introduction of US irradiation into the PDL group, and the stronger fluorescence in the PSDL group was observed. These data illustrate that PSDL may effectively boost the amount of ROS formation in cells, which is incredibly useful for enhancing the anticancer effect.

Phosphatidylserine (PS) externalization in cellular membranes is identified as a mechanism for inducing cell apoptosis [33]. Annexin V is a phospholipid-binding protein that can bind specifically to PS with high affinity during cell apoptosis. In order to verify whether cell apoptosis was induced in this progress, annexin V-FITC/PI was adopted for quantitative analysis. As plotted in Figure 3D, the apoptotic rates were about 2.13% in the control group. In addition, the PDL and PSDL alone group presented fractions of cell apoptosis of approximately 17.62% and 25.56%, respectively. When combined with US treatment, the proportion of apoptotic cells increased to 25.76% and 40.84% in the PDL group and PSDL group, respectively.

#### 2.3.3. DNA Damage

**Figure 4:**
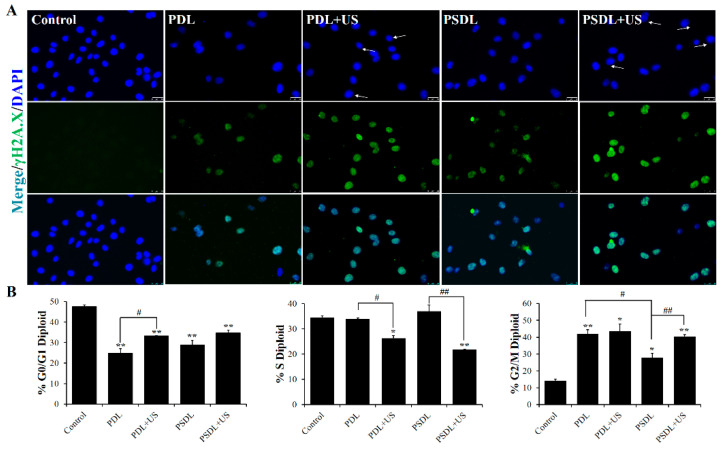
DNA damage analysis. (**A**) Immunofluorescence labeling γH2AX fluorescence intensity (scale bar = 25 μm); (**B**) the percentages of cells in each phase are plotted along the y-axis and the different treatments are graphed along the x-axis. * *p* < 0.05, ** *p* < 0.01, compared with control; ^#^ *p* < 0.05, ^##^ *p* < 0.01, compared among groups.

DNA damage is a trigger for apoptosis and can be induced by many factors, including ultraviolet/ionizing radiation [34,35], endogenous metabolites [36], environmental and dietary carcinogens [37], some anti-inflammatory drugs [38], and genotoxic anticancer therapeutics [39]. DNA damage may be classified into many categories, consisting of DNA strand breaks, abnormal DNA cross-linking, point mutations, and gene mutations. Double-strand breaks in DNA, a severe form of DNA damage, immediately result in phosphorylation of the core histone variation γH2A.X, which identifies the damaged DNA spot. The immunofluorescent staining test was adopted to determine the expression of the γH2A.X protein in this investigation. As illustrated in Figure 4A, compared to PDL plus US treatment, the strong fluorescence signal of phosphorylated-H2AX (γH2A.X) was observed in the PSDL+US group, indicating that serious DNA damage was caused and not repaired completely after PSDL+US treatment.

DNA damage at varying degrees may result in cellular senescence or transitory cell-cycle halt. Flow cytometry was utilized to investigate the cell cycle arrest in this research (Figure 4B). After 48 h of treatment, 14.3% of control cells accumulated in the G2/M. This increased to 42.09% and 43.62% in PDL group and PDL+US group, respectively. These results are consistent with the previous results showing that cell cycle fractions reveal a major G2/M phase arrest after DOX treatment compared to untreated cultures in C26 mouse colorectal adenocarcinoma cells [40], TK6 lymphoblastoid cells through inhibition of topoisomerase II [41], and MDA-MB-231 cells and nu/nu mice [42]. For the SRA737-loaded liposomes, the accumulation was as low as 27.79% and 40.55%, in PSDL group and PSDL+US group, respectively. Results showed that SRA737 could relieve MDA-MB-231 cells’ cell cycle arrest in the G2/M phase which was reduced by DOX. It is widely established that DNA damage generated by photoirradiation [43], radiotherapy [36], genotoxic agents [39], or even plant extract derivatives [44] may temporarily induce the arrest of the cell cycle, allowing time for repair. The G2/M checkpoint is critical for avoiding premature mitosis in cells with damaged DNA, particularly those lacking functioning p53 [45]. Interestingly, the MDA-MB-231 human breast cancer cell line has high levels of a mutant p53, so the arrest in the G2/M phase caused by the genotoxic agent DOX is readily comprehensible. After treatment with SRA737, the G2/M phase arrest was relieved, which may be attributed to the inhibited CHK1. As a master modulator of the DNA damage response, the regulation of cell cycle progression, repair of DNA damage, and DNA replication of CHK1 were all affected, thereby facilitating excessive damage of DNA and decreasing the fitness of overall survival of the tumor cells.

### 2.4. In Vivo Antitumor Effect

#### 2.4.1. Biodistribution and US-Triggered Drug Release In Vivo

**Figure 5:**
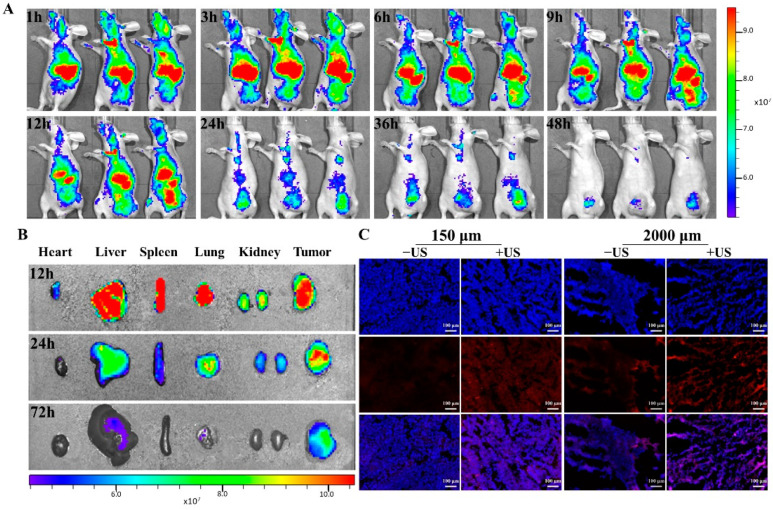
In vivo fluorescence imaging of PL in the orthotopic MDA-MB-231 tumor model. (**A**) Fluorescence imaging of PL in nude mice at different time points; (**B**) distribution of PL in the main organs and tumors of nude mice; (**C**) fluorescence microscope was used to observe the location and distribution of DOX in tumor tissue and cells with/without US irradiation (scale bar = 100 μm).

In this in vivo investigation, BALB/c nude mice were xenografted with MDA-MB-231 tumors. Figure 5A illustrated that tumors exhibited gradually increased porphyrin fluorescence of PL until 12 h, and then it declined at 24 h, illustrating that PL may have aggregated in the tumor via increased permeability along with retention. Additionally, the PL biodistribution in each excised organ was assessed at several time intervals (12 h, 24 h, and 72 h) after liposome injection (Figure 5B). At 24 h, the fluorescence intensity in tumor tissue was much greater than that in normal organs (heart, lung, liver, spleen, and kidney).

Consequently, the location and distribution of DOX in tumor tissue were analyzed (Figure 5C and Supplementary Figure S4). Results showed an enhanced and dispersed pattern of DOX fluorescence following US exposure, as well as DOX fluorescence penetrating into cell nuclei. In comparison, sections of tumors that had not been exposed to US demonstrated weaker fluorescent signals that were seldom found in tumor cell nuclei.

#### 2.4.2. Antitumor Efficacy

**Figure 6:**
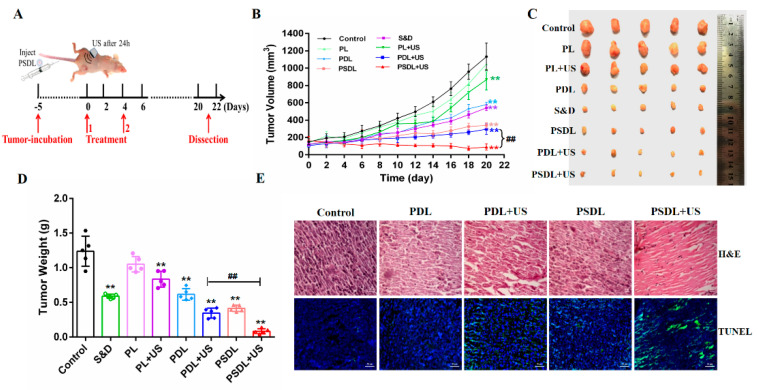
Antitumor assessment in vivo. (**A**) Schematic illustration of sequence of treatment in vivo; (**B**) tumor growth curves of MDA-MB-231 tumor-bearing nude mice after different treatments; (**C**) tumor photos of different groups; (**D**) average tumor weight inhibition ratio of mice in each treatment group; (**E**) H&E staining in MDA-MB-231 tumors (scale bar = 20 μm) and TUNEL staining in MDA-MB-231 tumors (scale bar = 50 μm). ** *p* < 0.01, compared with control; ^##^ *p* < 0.01, compared among groups.

MDA-MB-231 cells were implanted into nude mice in situ to assess the antitumor effectiveness. To explore the anticancer efficacy of diverse liposomes coupled with US in vivo, tumor-harboring mice were stratified into eight study groups. In detail, the different liposomes were i.v. injected to mice (Figure 6A). First, the harvested tumors and their volume and weight were measured (Figure 6B–D). The group treated with PL exhibited no remarkable change from the control group. On the 20nd day after treatment, the tumor volume suppressing ratios for the S&D, PL, PDL, PL+US and PDL+US groups were 53.59%, 10.21%, 49.14%, 25.19% and 74.24%, respectively, as shown in Figure 6B. Notably, the ratio of tumor volume inhibition was unexpectedly as high as 69.36% and 92.21% in PSDL and PSDL+US groups, respectively. As expected, a higher ratio of tumor weight suppression was also detected, analogous to the result of tumor volume suppression (Figure 6D). These findings imply that adding SRA737 to DOX-loaded Pp18 liposomes enhances their anticancer efficacy in vivo.

Further, to establish PSDL’s superior effectiveness in assessing anticancer activity, tumor tissue slices were stained with H&E. In comparison to other groups, evident tissue damage with big spaces was observed after PSDL+US therapy. The finding concurred with the TUNEL experiment data (Figure 6E), which established that PSDL+US triggered remarkable cell death, hence dampening tumor development. 

**Figure 7:**
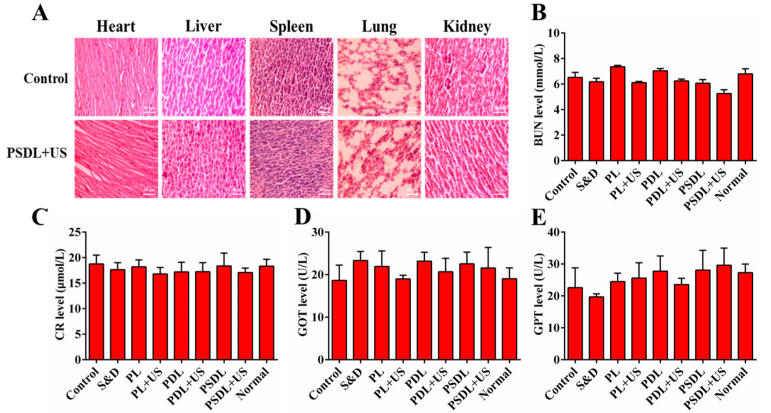
Evaluation of side effects. (**A**) H&E staining of heart, liver, spleen, and kidney tissues of mice after different treatments (scale bar = 50 μm); (**B**) BUN, (**C**) CR, (**D**) GOT, and (**E**) GPT content analysis of tumor-bearing mice.

Additionally, no apparent toxicity (as demonstrated, for instance, by a steady body weight) was observed in the course of the research period (Supplementary Figure S5). In addition, tissue slices from important organs (liver, lung, spleen, and kidney) were also obtained. H&E staining (Figure 7A) along with blood biochemical index analysis (Figure 7B–E) both exhibited no pathological differences in each treatment group. These findings established that PSDL administration did not cause substantial systemic toxicity in the treated animals and seemed to be safe throughout the investigation.

## 3. Materials and Methods

### 3.1. Materials

1,2-Distearoyl-sn-glycero-3-phosphocholine (DSPC), cholesterol (Chol), 1-palmitoyl-2-hydroxy-sn-glycero-3-phosphocholine (P-lyso PC), and 1,2-distearoyl-sn-glycero-3-phosphoethanolamine-N-[amino(polyethylene glycol)-2000] (DSPE-PEG2000) were provided by Xi’an Ruixi Biological Technology Co., Ltd. (Xi’an, China). Purpurin 18 (Pp18) was supplied by Xianhui Pharmaceutical Co., Ltd. (Shanghai, China). 1-(3-Dimethylaminopropyl)-3-ethylcarbodiimide hydrochloride (EDC) and 4-(dimethylamino) pyridine (DMAP) were acquired from Aladdin (Shanghai, China). DOX and SRA737 were purchased from Sigma-Aldrich (St. Louis, MO, USA) and MedChemExpress (Monmouth Junction, NJ, USA), respectively. 2,7-Dichlorodihydrofluorescein diacetate (DCFH-DA) and Hoechst 33,258 were purchased from Invitrogen (Carlsbad, CA, USA). Calcein-AM/PI Double Staining Kit was provided by Yeasen (Shanghai, China). Cell Counting Kit-8 kit (CCK-8) was supplied by Beyotime (Shanghai, China).

### 3.2. Cell Line and Animal Model

The MDA-MB-231 human breast cancer cells were acquired from the Cell Resource Center of the Chinese Academy of Science, China. The cells were inoculated in DMEM medium enriched with 10% FBS. The female BALB/c mice (female, 18–20 g and 6–8 weeks) were obtained from the Animal Centre of Xi’an Jiao Tong University (Xi’an, China). Mice were housed in isolated cages in a 12 h light/dark cycle environment. After one-week acclimation, the mice were inoculated with MDA-MB-231 cells (3 × 10^6^ cells/mL) in situ. The tumor-harboring mice were assigned at random into diverse treatment groups.

### 3.3. Synthesis and Characterization of PSDL Nanoparticles

#### 3.3.1. Pp18-Liposome (PL) Synthesis

Pp18–lipid conjugates were firstly prepared via an acylation reaction between Pp18 and P-lyso PC under catalysis of EDC and DMAP as previously described [15]. On the basis of that, the Pp18-liposomes synthesis began with the thin film hydration method. The film consisted of 58 mol% DSPC, 1 mol% DOPC, 5 mol% DSPE-PEG2000, 35 mol% cholesterol, and 1 mol% Pp18-lipid. The dried film was hydrated with 1 mL 0.01 M PBS (pH 7.0~7.2) and then subjected to eight freeze–thaw cycles. The liposomes were then extruded stepwise though polycarbonate membranes with pore sizes of 200 and 100 nm at 65 °C with a mini-extruder (Avanti Polar Lipids. Inc. Alabaster, AL, USA) to afford PL.

#### 3.3.2. SRA737/DOX Co-Loaded Pp18-Liposome (PSDL) Synthesis

SRA737 loading was achieved by addition to a lipid mixture before film-forming. In addition, DOX was added to hydrated liposomes in a 1:20 weight ratio of drug:lipid and incubated for 1 h at 65 °C. The free SRA737/DOX was removed by dialysis.

UV absorption/fluorescence was adopted to determine the entrapment effectiveness of SRA737/DOX via a multimode microplate reader (Tecan, Männedorf, Switzerland). The polydispersity index, mean size, and zeta potential of distinct liposome groups were measured with a Malvern Zetasizer Nano instrument (ZS90, Malvern, UK).

#### 3.3.3. PSDL Nanoparticle Release Test

PSDLs were inoculated with diverse ultrasonic intensities and durations (load power: 1 W, 2 W, 3 W; duration 30 s, 60 s, 120 s) before being moved into a dialysis tube with a molecular weight cutoff (MWCO) of 3500 Da. The sealed dialysis tube was submerged in 30 mL of PBS solution and gently agitated at 37 °C for 0.5~24 h. For UV fluorescence analysis, samples of 0.3 mL dialysate were obtained at 0, 0.5, 1, 2, 3, 4, 8, 12, and 24 h.

### 3.4. In Vitro Antitumor Effect of PSDL Combined with US

#### 3.4.1. In Vitro Cell Uptake and Cytotoxicity

MDA-MB-231 cells were inoculated with diverse liposome groups to assess cellular uptake using the intracellular DOX fluorescence. Moreover, the influence of low-intensity ultrasound (load power: 2 W; duration 30 s) on different liposomes was also investigated. After 4 h, cells were rinsed twice with PBS, counter-stained with DAPI, and observed using a confocal microscope (Model TCS SP8, Leica, Wetzlar, Germany).

MDA-MB-231 cells (1 × 10^4^ cells/well) were plated in a 96-well plate for in vitro cytotoxic tests. For 12 h, different dosages of SRA737/DOX in free and liposomal forms were introduced. The wells were then irradiated with the indicated US treatment. After 24 h, the CCK-8 test was utilized to assess the cell cytotoxicity of various drugs. Calcein AM/PI was also used to test cell viability following PBS, PL, PDL, and PSDL with or without US treatments.

#### 3.4.2. Cell Apoptosis

The annexin V-FITC/PI double staining kit was adopted to assess the apoptosis as described by the manufacturer. Cells from each group were trypsinized, collected, and rinsed twice in sterile PBS. The cells were then re-suspended in 500 μL of binding buffer, stained with 5 μL of annexin V-FITC and 5 μL of PI, and inoculated at room temperature (RT) for 15 min before being analyzed via flow cytometry. Apoptotic cells were defined as annexin V-FITC^+^/PI^-^ and annexin V-FITC^+^/PI^+^ cell populations.

#### 3.4.3. Assessment of DNA Damage In Vitro

The SDT can not only kill cells directly, but it can also destroy them indirectly by damaging DNA molecules [46]. The phosphorylation of γH2A.X increases after exposure to DNA-damaging chemicals, and it is often utilized as a double-strand break marker. The degree of phosphorylation of γH2AX was determined by immunofluorescence, as reported before with some modifications [47]. Primary antibodies against p-γH2AX were incubated with cells overnight at 4 °C, followed by matching secondary antibodies conjugated with Dylight 488 for 1 h at 37 °C. Finally, the cells were exposed to Hoechst 33342 (10 M) for 15 min before being examined under a fluorescence microscope (Nikon, Tokyo, Japan).

Multiple signal transduction cascades are activated by DNA damage, resulting in growth arrest along with responses of DNA damage repair. Cells may repair their DNA before mitosis by interrupting the cell cycle after DNA damage. To analyze the change in cell cycle, MDA-MB-231 cells (1 × 10^4^ cells/well) were plated in a 96-well plate for 12 h, and then different dosages of SRA737/DOX in free and liposomal forms were introduced. The wells were then irradiated with the US treatment indicated. At 48 h after US treatment, the obtained cells were extracted and treated as follows to check whether growth arrest along with DNA damage repair responses transpired after inoculation with the various liposomes: rinsed twice with cold PBS enriched with 1%BSA, fixed overnight with 70% ice-cold ethanol at −20 °C, rinsed twice with cold PBS, inoculated for 30 min with 100 μg/mL RNase A at 37 °C, stained in the dark with 50 μg/mL PI for 30 min, and analyzed (10,000 cells/sample) via flow cytometry (ACEA NovoCyte, San Diego, CA, USA).

#### 3.4.4. Intracellular ROS Generation

MDA-MB-231 cells (1 × 10^4^ cells/well) were plated in a 96-well plate for intracellular ROS tests. Briefly, cells were incubated with 10 μM DCHF-DA at 37 °C for 30 min prior to US treatment. At 1 h after US treatment, the cells were washed with PBS and imaged using a fluorescence microscope.

### 3.5. Antitumor Effect of PSDL Nanoparticles Combined with US In Vivo

#### 3.5.1. In Vivo Fluorescence Imaging

MDA-MB-231 tumor-bearing mice were adopted to evaluate the biodistribution of PL. When the volume of the tumors reached 100–150 mm^3^, the tumor-harboring mice were inoculated with PL (1.57 mg/kg Pp18) through the tail vein. The fluorescence pictures were then acquired using an IVIS spectrum small animal imaging system (PerkinElmer, Norwalk, CT, USA) with excitation along with emission wavelengths of 710 nm and 780 nm, respectively. After PL injection, the mice were scanned at 1 h, 3 h, 6 h, 9 h, 12 h, 24 h, 36 h, and 48 h. Mice were sacrificed at 12 h, 24 h, 72 h, respectively, after inoculation to assess the primary organs, including the heart, liver, spleen, lung, kidney, and tumor.

#### 3.5.2. Ex Vivo Fluorescence Imaging

Tumor-harboring mice were inoculated with PDL (5 mg/kg DOX) through the tail-veil to assess DOX biodistribution in response to US exposure. Tumors were subjected to US intensity after 24 h. The mice were subsequently sacrificed, and the tumors were frozen in an OCT gel before being sectioned and examined via a fluorescence microscope. Afterward, the frozen slides were stained with DAPI, and fluorescence microscopy was adopted to determine the subcellular distribution of DOX.

#### 3.5.3. In Vivo Antitumor Studies

BALB/c nude mice were xenografted with MDA-MB-231 cells in situ to assess antitumor effectiveness. After 1–2 weeks of tumor development, the tumor volumes were around 100–150 mm^3^ and suitable for analyses. Liposomes were triggered using US at 24 h after diverse administrations. Vernier calipers were utilized to measure the lengths along with widths of tumors, and the tumor volume was estimated using the following equation: tumor volume (mm^3^) = (length ×width × width)/2. To assess tumor damage, a histopathological investigation was performed, and tumor sections were stained with H&E. The apoptosis of cells was investigated using the TUNEL staining test.

### 3.6. Statistical Analysis

The experimental data were assessed using one-way analysis of variance (ANOVA) or Student’s t-test, and multiple comparisons between groups were performed using Tukey’s test. Statistical significance was denoted at *p* < 0.05 (* or ^#^), while *p* < 0.01 (** or ^##^) was highly significant.

## 4. Conclusions

To address fundamental shortcomings of conventional cancer treatment methods, we designed SRA737-DOX@Plipo nanocomposites that could be activated by US for an enhanced antitumor therapy through integrating chemotherapy with SDT. Its antitumor activity was investigated in vitro along with in vivo. To begin, drug loading and release investigations established that the nanoparticles were of a suitable size and had a rather high drug loading capacity. In vitro and in vivo studies both exhibited that PSDL combined with US was more effective against tumors relative to PDL+US or PSL+US alone. As a result, we hypothesized that after the drug-loaded Plipo nanoparticles reached the bloodstream, PSDL passively targeted and enriched tumor tissue through the EPR effect, releasing SRA737 along with DOX and increasing the drug concentration around the cancer cells. Upon sonication activation, Pp18-mediated SDT exhibited anticancer activity in combination with SRA737 and DOX-mediated chemotherapy; thus, PSDL exhibited greater antitumor activity in comparison to that of the other groups. In addition, our findings illustrated that combining chemotherapy with SDT is a relatively safe and effective strategy with good therapeutic promise for breast cancer.

## Data Availability

The data that support the findings of this study are available from the corresponding author upon reasonable request.

## Supplementary Materials

The following supporting information can be downloaded at: https://www.mdpi.com/article/10.3390/ijms23147981/s1.
