# Supplementary material for: Mechanism-Based Sonodynamic–Chemo Combinations against Triple-Negative Breast Cancer
Source: Int J Mol Sci. 2022 Jul 20;23(14):7981. doi: 10.3390/ijms23147981 (PMC9315679; doi:10.3390/ijms23147981)
Supplement: Supplementary file 1 [file ijms-23-07981-s001.zip › ijms-1791119-supplementary.pdf]

Supporting information

Mechanism-Based Sonodynamic-Chemo Combinations against Triple-Negative Breast Cancer

Xiaolan Feng[1,2,†], Chen Wu[1,2,†], Wenhao Yang[1,2], Jiayi Wu[1,2], Pan Wang[1,2,*]

[1] Key Laboratory of Medicinal Resources and Natural Pharmaceutical Chemistry, Ministry of Educa-tion;,

[2] National Engineering Laboratory for Resource Developing of Endangered Chinese Crude Drugs in Northwest of China, College of Life Sciences, Shaanxi Normal University, Xi'an 710119, China.

* Correspondence: wangpan@snnu.edu.cn; Tel.: +86-029-85310275.

† These authors contributed equally to this work.

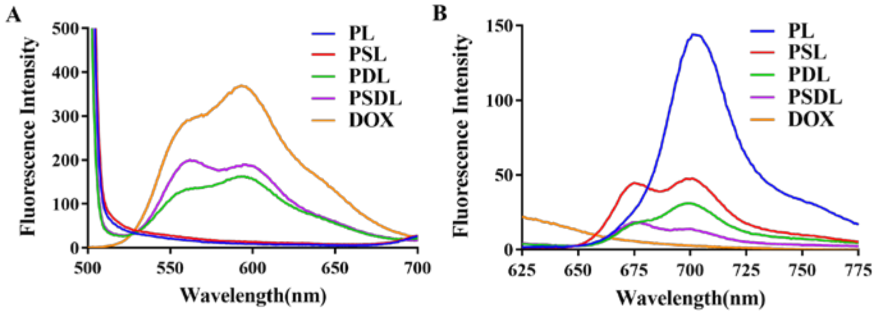

Figure S1. The fluorescence emission spectra of DOX (A, 480 nm excitation) and Pp18 (B, 410 nm excitation).

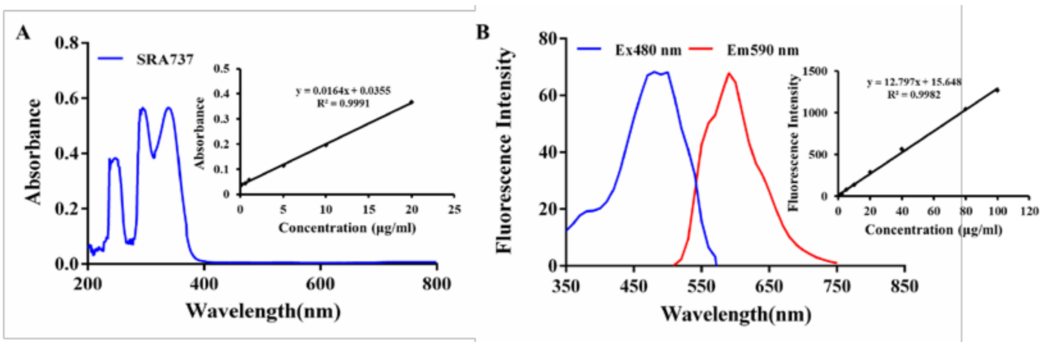

Figure S2. The spectrum and standard curve. (A) Absorption spectrum and standard curve of SRA737; (B) Excitation-Emission spectroscopy and standard curve of DOX.

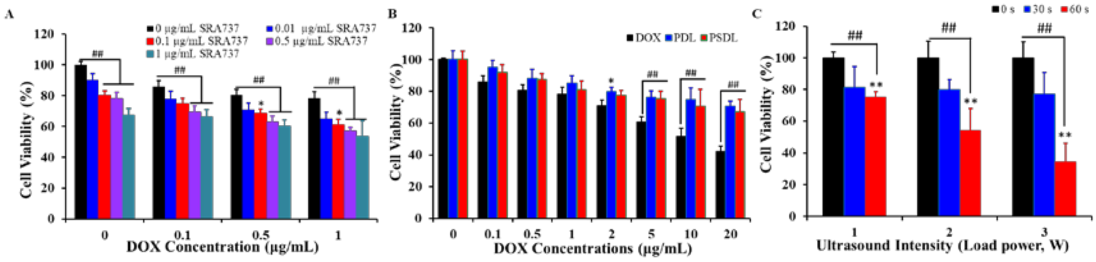

Figure S3. Cytotoxicity evaluation in vitro. (A) The effects of different concentrations of DOX combined with SRA737 on MDA-MB-231 cells viability; (B) The effect of different treatments

on the cell viability; (C) Different ultrasonic intensity and duration time on cell survival rate.

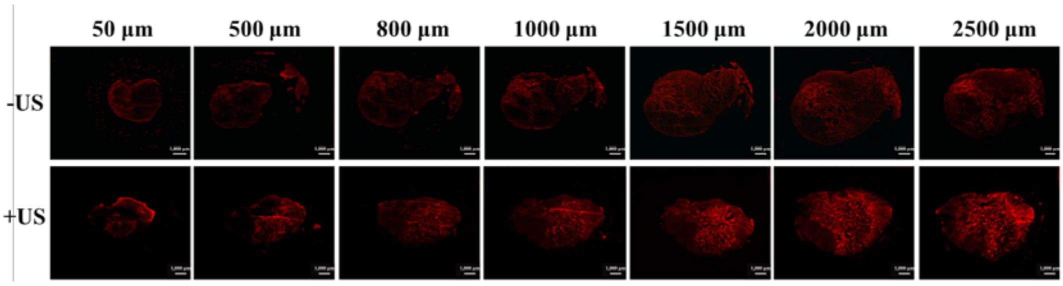

Figure S4. Continuous frozen sections were used to detect the penetration of PDL in tumor tissues with or without US using the red fluorescence of emission DOX. Bar = 1000 μm.

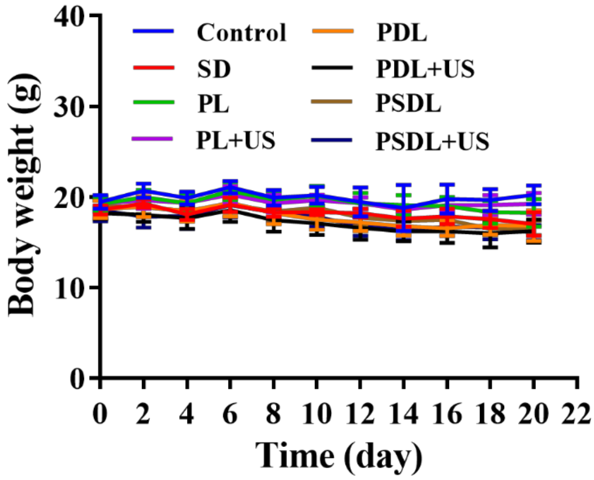

Figure S5. The body weight of MDA-MB-231 tumor-bearing nude mice after different treatments.

Table S1. Drug Loading Properties of PSL

SRA737: P-Lipos	Mean diameter (nm)	PDI	Encapsulation efficiency (%)	Loading efficiency (%)
1: 100	106.5±3.80	0.030±0.03	19.33%±3.22	0.19%±0.03
1:50	116.6±5.78	0.035±0.05	35.77%±4.12	0.70%±0.08
1:25	124.6±3.44	0.030±0.03	26.60%±0.92	1.02%±0.04
1:20	128.4±3.54	0.068±0.03	29.31%±2.15	1.40%±0.10

Table S2. Drug Loading Properties of PDL

DOX: P-Lipos	Mean diameter (nm)	PDI	Encapsulation efficiency (%)	Loading efficiency (%)
1:50	119.3±3.82	0.103±0.07	45.33%±4.55	0.89%±0.09
1:25	123.9±1.92	0.064±0.05	60.78%±4.11	2.34%±0.16
1:20	126.8±5.27	0.042±0.04	67.60%±3.46	3.22%±0.16
1:10	132.5±4.31	0.067±0.06	49.90%±2.46	4.54%±0.22

Table S3. The mean diameter, PDI and encapsulation efficiency in different nanoparticles.

Sample	Mean Diameter (nm)	PDI	Encapsulation efficiency (%)-SRA737	Encapsulation efficiency (%)-DOX
PL	114.7±3.49	0.095±0.06	--	--
PSL	125.8±2.60	0.049±0.03	35.77%±4.12	--
PDL	126.9±3.46	0.041±0.04	--	68.85%±4.10
PSDL	132.7±3.10	0.036±0.04	31.34%±3.52	60.52%±5.65
